# The Burden of Respiratory Abnormalities Among Workers at Coffee Roasting and Packaging Facilities

**DOI:** 10.3389/fpubh.2020.00005

**Published:** 2020-01-30

**Authors:** R. Reid Harvey, Ethan D. Fechter-Leggett, Rachel L. Bailey, Nicole T. Edwards, Kathleen B. Fedan, M. Abbas Virji, Randall J. Nett, Jean M. Cox-Ganser, Kristin J. Cummings

**Affiliations:** Respiratory Health Division, National Institute for Occupational Safety and Health, Centers for Disease Control and Prevention, Morgantown, WV, United States

**Keywords:** coffee roasting and packaging, occupational asthma, obliterative bronchiolitis, flavoring, diacetyl, 2, 3-pentanedione, coffee dust

## Abstract

**Introduction:** Respiratory hazards in the coffee roasting and packaging industry can include asthmagens such as green coffee bean and other dust and alpha-diketones such as diacetyl and 2,3-pentanedione that can occur naturally from roasting coffee or artificially from addition of flavoring to coffee. We sought to describe the burden of respiratory abnormalities among workers at 17 coffee roasting and packaging facilities.

**Methods:** We completed medical surveys at 17 coffee roasting and packaging facilities that included interviewer-administered questionnaires and pulmonary function testing. We summarized work-related symptoms, diagnoses, and spirometry testing results among all participants. We compared health outcomes between participants who worked near flavoring and who did not.

**Results:** Participants most commonly reported nose and eye symptoms, and wheeze, with a work-related pattern for some. Symptoms and pulmonary function tests were consistent with work-related asthma in some participants. About 5% of workers had abnormal spirometry and most improved after bronchodilator. Health outcomes were similar between employees who worked near flavoring and who did not, except employees who worked near flavoring reported more chronic bronchitis and ever receiving a diagnosis of asthma than those who did not work near flavoring.

**Conclusion:** The symptoms and patterns likely represent overlapping health effects of different respiratory hazards, including green coffee bean and other dust that can contribute to work-related asthma, and diacetyl and 2,3-pentanedione that can contribute to obliterative bronchiolitis. Healthcare providers and occupational health and safety practitioners should be aware that workers at coffee roasting and packaging facilities are potentially at risk for occupational lung diseases.

## Introduction

Five cases of obliterative bronchiolitis were diagnosed among former workers of a U.S. coffee roasting and packaging facility during 2012–2015; two cases were confirmed by lung biopsy (1, 2). This cluster of obliterative bronchiolitis was the first identified among workers in the coffee roasting and packaging industry. Obliterative bronchiolitis (also called bronchiolitis obliterans or constrictive bronchiolitis) is a rare and irreversible lung disease characterized by inflammation and fibrotic changes leading to narrowing of the small airways (<2 mm, bronchioles) (3). Symptoms often include cough, exertional dyspnea, or wheeze, typically without a work-related pattern (4). Occupational obliterative bronchiolitis was described in 2002 among workers at a microwave-popcorn production facility that used artificial butter flavoring containing diacetyl (5, 6). Investigations at other microwave popcorn production facilities and in flavoring and food manufacturing facilities that used or produced flavorings containing diacetyl identified additional cases of flavoring-related obliterative bronchiolitis (7–9). Subsequent experimental studies revealed inhalational exposure to diacetyl, caused severe injury to the respiratory epithelium in animals (10–13). Animal studies also demonstrated another closely related compound, 2,3-pentanedione, causes similar toxicity and should therefore not be considered a potential safe substitute for diacetyl by industry (14–16).

The sentinel coffee facility that had employed the former workers who had obliterative bronchiolitis added flavorings that contained the alpha-diketones diacetyl and 2,3-pentanedione to coffee in a separate, enclosed area of the facility; however, diacetyl and 2,3-pentanedione are also naturally produced and released during the coffee roasting process (17). An industrial hygiene investigation based on alpha-diketone levels measured during grinding, packaging, and off-gassing of unflavored roasted coffee, determined sources of diacetyl and 2,3-pentanedione were not restricted to the areas of the facility where flavorings were added (18). Additionally, more workers than expected at the sentinel coffee facility had exertional dyspnea and spirometric obstruction, but not all of these workers were located in the flavoring area of the facility (1). The investigation suggested that natural sources of diacetyl and 2,3-pentanedione might contribute to respiratory disease risk in the coffee roasting and packaging industry, in addition to the known risk from added flavorings.

Workers in the coffee roasting and packaging industry are susceptible to other work-related respiratory diseases in addition to obliterative bronchiolitis, most notably work-related asthma (19). Work-related asthma encompasses both incident occupational asthma and exacerbation of pre-existing asthma (20–22). Symptoms often include shortness of breath, cough, wheeze, or chest tightness that frequently improve away from work. Green and roasted coffee dust, and castor bean dust from contaminated burlap bags used to ship green coffee beans, are established causes of work-related asthma in coffee roasting and packaging (19, 23–27). Work-related asthma can be caused by different mechanisms, including an allergic response to sensitizers like green coffee beans or a non-allergic, irritant induced response to coffee dust (28).

During 2016–2017, the U.S. National Institute for Occupational Safety and Health (NIOSH) evaluated an additional 17 coffee roasting and packaging facilities to address concerns about workplace exposures to diacetyl and 2,3-pentanedione, and other potential respiratory hazards like green coffee beans and dust. Some of the facilities added flavorings to roasted coffee, and others did not. No cases of obliterative bronchiolitis or severe lung disease among workers at these coffee roasting and packaging facilities had been identified prior to our evaluations. We present the combined health evaluations from 17 facilities to describe the burden of respiratory abnormalities among their coffee roasting and packaging workers.

## Methods

During 2016–2017, NIOSH responded to 17 management or employee requests for health hazard evaluations (https://www.cdc.gov/niosh/hhe/default.html) at coffee roasting and packaging facilities to primarily address concerns about potential exposure to diacetyl and 2,3-pentanedione. Each facility was evaluated independently and received its own report of findings and recommendations (available at: https://www2a.cdc.gov/hhe/search.asp). We will present detailed results of the industrial hygiene surveys assessing diacetyl and 2,3-pentanedione in these 17 facilities separately. The NIOSH Institutional Review Board approved this study that pools the data from those 17 public health evaluations (NIOSH Protocol 17-RHD-06XP). All current workers aged 18 years or older at the coffee roasting and packaging facilities were invited to give written informed consent for an evaluation that included an interviewer-administered questionnaire, spirometry, and exhaled nitric oxide. The questionnaire addressed symptoms, diagnoses, work history, work-tasks and exposures, smoking history, and demographic information. Respiratory symptom questions were adapted from validated survey instruments (29–35). We defined work-related symptoms as those reported to be better away from work. For current or former smokers, we calculated smoking pack-year as 20 cigarettes smoked per day for 1 year.

We used a volume spirometer, American Thoracic Society (ATS) criteria for acceptability and repeatability of spirometry tests, and equations for predicted values and lower limits of normal derived from the Third National Health and Nutrition Examination Survey (NHANES) data to define abnormal spirometry (35–37). We defined obstruction as a forced expiratory volume in 1 s (FEV_1_)/forced vital capacity (FVC) ratio and FEV_1_ less than their respective lower limits of normal (LLN); restrictive pattern as an FVC less than the LLN with normal FEV_1_/FVC ratio; and mixed obstruction and restrictive pattern as having FVC and FEV_1_/FVC ratio less than their respective LLNs. We used the FEV_1_ percent predicted to categorize abnormalities as mild, moderate, moderately severe, severe, or very severe (38). All participants with abnormal spirometry were offered bronchodilator testing to assess for reversibility of at least 12% and 200 milliliters (mL) for either FEV_1_ or FVC with albuterol as the bronchodilator. We used the NIOX MINO® device (Aerocrine Inc., Morrisville, NC) to measure fractional exhaled nitric oxide (FeNO). FeNO concentrations above 50 parts per billion (ppb) were considered elevated (39).

We used participants' narrative descriptions of how work causes or aggravates upper respiratory symptoms (nasal symptoms or sinus problems) and lower respiratory symptoms (wheeze, exertional dyspnea, breathing trouble, cough, chest tightness, asthma attack, or awoken by shortness of breath) to create word clouds using R 3.5.2 (R Core Team, 2019) and the *wordcloud* (v2.6; Fellows, 2018) package. Words or short phrases were sized proportionally to the frequency used to provide graphic representations of keywords used by participants to describe causes or aggravations of symptoms at work.

We compared symptoms, diagnoses, and lung function parameters between participants who reported working near flavoring (within an arm's length of the container when flavorings are being added or mixed with roasted coffee) and participants who did not report working near flavoring. We compared symptoms, diagnoses, and lung function parameters between atopic participants (those with self-reported hay fever, nasal allergies, or eczema) and non-atopic participants. We calculated chi-square values to compare health outcomes between participants who reported working near flavoring and those who did not, and participants who reported atopy and those who did not. We considered *P* < 0.05 to be statistically significant.

We calculated frequencies and standardized morbidity ratios (SMRs) and their associated 95% confidence intervals (CIs). The SMRs compared prevelences of symptoms, diagnoses, and spirometric abnormalities among participants with expected prevelences of a sample of the general U.S. population reflected in NHANES data, adjusting for race/ethnicity (white, black, Hispanic), sex, age (≤39 and ≥40 years), and smoking (ever/never) (34, 35). We used the most recent NHANES data available for the specific comparisons, including NHANES III (1988–1994) and NHANES Continuous (select years during 1999–2016).

We compared our study participants to several previous study populations, including the sentinel coffee roasting and packaging facility, sentinel microwave popcorn production facility, and combined data from three other microwave popcorn facilities (1, 7, 18, 40–43). For these comparisons, we categorized the 17 facilities included in our study in to two groups—those that used flavoring or those who did not (non-flavoring). We compared exertional dyspnea, wheeze, cough, percent predicted FEV_1_, obstruction, and mean time weighted average (TWA) personal exposure to diacetyl and 2,3-pentanedione. All statistical analyses were conducted using SAS version 9.4 (SAS Institute Inc., Cary, NC).

## Results

We evaluated 384 (58%) of 658 current workers from 17 coffee roasting and packaging facilities in 12 states during 2016–2017. The facilities had a median of 15 participating workers (range: 4–99 workers). Most participants were male (59%) and white (59%), with a median age of 35 years (range: 18–72 years) (Table 1). Most participants were never smokers (57%); 43% were current or former smokers with a median of 3.3 pack-years. The median number of years worked at the current facility (tenure) was 2.8 (range: <1–30 years); 79 (21%) participants reported previously working at other coffee roasting and processing facilities or companies that use flavorings, and the median number of years worked at any coffee facility or company that uses flavorings was 3.5 (range: <1–34 years).

**Table 1:** Characteristics and job tasks of participating workers in 17 coffee roasting and production facilities, *N* = 384.

**Characteristic**	***n* (%)**
Age (years) median (range)	35 (18–72)
**Sex**	
Male	225 (59%)
Female	159 (41%)
**Race/ethnicity**	
Non-hispanic white	226 (59%)
Hispanic	112 (29%)
Black	29 (8%)
Asian	7 (2%)
Other, including multi-racial	10 (3%)
**Body mass index**	
BMI ≥ 30	125 (33%)
**Smoking status**	
Never	220 (57%)
Former	98 (26%)
Current	66 (17%)
**Tenure at current facility (years) median (range)**	2.8 (<1–30)
**Job tasks**	*n* (%)
Package coffee	211 (55%)
Move coffee	185 (48%)
Clean equipment	177 (46%)
Grind coffee	162 (42%)
Perform maintenance	105 (27%)
Roast coffee	60 (16%)
Flavor coffee	23 (6%)

Most participants (87%) reported currently working in production areas of a coffee roasting and processing facility where the most common tasks performed included packaging coffee (55%), moving coffee (48%), cleaning equipment (46%), and grinding coffee (42%) (Table 1). Sixty (16%) participants roasted coffee. Of the 17 coffee roasting and packaging facilities, 12 did not flavor coffee, while the other five did flavor some of the coffee processed in their facility. At the five facilities that flavored coffee, 23 (16%) of 143 participants who worked in production reported performing the task of flavoring coffee. Eleven (65%) of 17 facilities also included a café; 35 (23%) of 149 participants from facilities with cafés reported working in the cafés, although these employees could have also had other job responsibilities, including production tasks.

**Figure 1:**
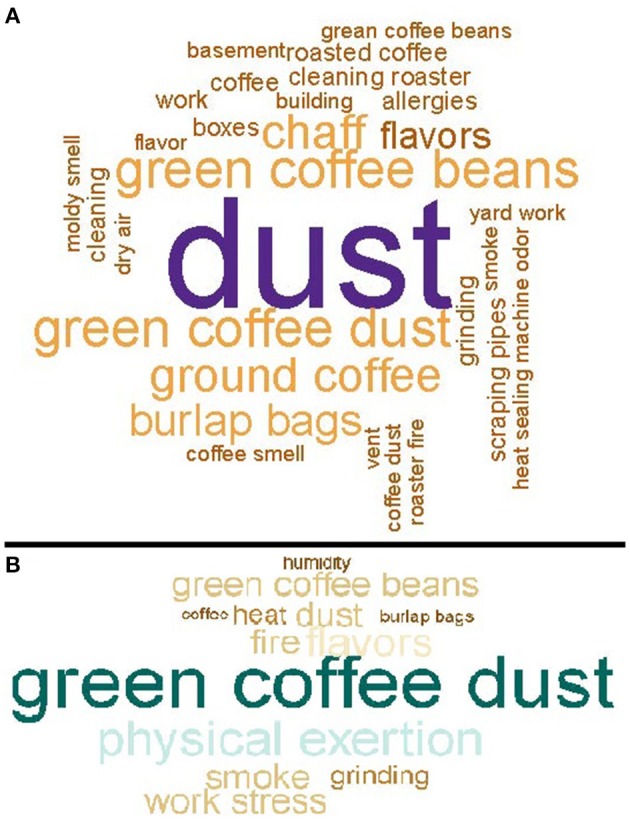
**(A)** Causes or aggravations of upper respiratory symptoms at work, for workers in 17 coffee roasting and production facilities, *N* = 163. **(B)** Causes or aggravations of lower respiratory symptoms at work for workers in 17 coffee roasting and production facilities, *N* = 39.

Upper respiratory symptoms in the last 12 months were the most commonly reported symptoms (66% of participants); 11% of participants reported their upper respiratory symptoms were work-related (Table 2). Participants who reported their upper respiratory symptoms were caused or aggravated by work most commonly implicated dust [55 of (34%) 163 respondents] (Figure 1A). Compared with the U.S. adult population, participants were more likely to report a stuffy, itchy, or runny nose in the last 12 months (SMR 1.2; 95% CI: 1.1–1.4) (Table S1). Eye symptoms in the last 12 months were reported by 49% of participants; 11% of participants reported their eye symptoms were work-related. Compared with the U.S. adult population, participants were more likely to report watery, itchy eyes in the last 12 months (SMR 1.2; 95% CI: 1.0–1.4).

**Table 2:** Prevalence of reported symptoms and work-relatedness, and self-reported doctor diagnoses and diagnoses post-hire, by workers in 17 coffee roasting and production facilities, *N* = 384.

**Symptom(s)**	**Last 12 months *n* (%)**	**Last 4 weeks *n* (%)**	**Work-related *n* (%)**
Upper respiratory symptoms (reported at least one of the following)	252 (66%)	152 (40%)	41 (11%)
Nose symptoms^*^	244 (64%)	145 (38%)	35 (9%)
Sinusitis or sinus problems	105 (27%)	50 (13%)	13 (3%)
Eye symptoms^†^	187 (49%)	117 (30%)	44 (11%)
Problem with ability to smell	46 (12%)	-	-
Phlegm on most days for 3 months	40 (10%)	-	-
Lower respiratory symptoms (reported at least one of the following)	179 (47%)	82 (21%)	39 (10%)
Chest wheezing or whistling	94 (24%)	36 (9%)	15 (4%)
Exertional dyspnea^‡^	59 (15%)	-	-
Breathing trouble	79 (21%)	45 (12%)	17 (4%)
Awoke with chest tightness	53 (14%)	13 (3%)	14 (4%)
Usual cough^§^	40 (10%)	27 (7%)	8 (2%)
Awoke with shortness of breath	28 (7%)	8 (2%)	2 (1%)
Asthma attack	26 (7%)	9 (2%)	2 (1%)
Systemic symptoms (reported at least one of the following)	199 (52%)	94 (24%)	50 (13%)
Flu-like achiness or achy joints	145 (38%)	50 (13%)	17 (4%)
Fever or chills	99 (26%)	22 (6%)	11 (3%)
Unusual tiredness or fatigue	88 (23%)	60 (16%)	30 (8%)
**Diagnosis**	***n*** **(%)**	**Post-hire** ***n*** **(%)**	
Hay fever or nasal allergies	88 (23%)	17 (4%)	
Eczema	47 (12%)	10 (3%)	
Heart disease	11 (3%)	6 (2%)	
Gastroesophageal reflux disease	30 (8%)	12 (3%)	
Chronic bronchitis	6 (2%)	1 (<1%)	
Asthma (ever)	65 (17%)	6 (2%)	
Asthma (current)	38 (10%)	6 (2%)	

*“-” = A 4 week question or work-related question was not asked for the symptom*.

* *Nose symptoms includes one or both of the following: (1) stuffy, itchy, or runny nose or (2) stinging, burning nose*.

† *Eye symptoms includes one or both of the following: (1) watery, itchy eyes or (2) stinging, burning eyes*.

‡ *This question did not specifically ask about exertional dyspnea within the past 12 months; participants were asked, “Are you troubled by shortness of breath when hurrying on level ground or walking up a slight hill”*.

§ This question did not specifically ask about a cough within the past 12 months; participants were asked, “Do you usually have a cough?” If the participants answered “yes,” they were then asked, “Have you had a cough at any time in the last 4 weeks?”

Lower respiratory symptoms in the last 12 months were reported by 47% of participants; 10% of participants reported their lower respiratory symptoms were work-related (Table 2). Participants who reported their lower respiratory symptoms were caused or aggravated by work most commonly implicated green coffee dust [8 (21%) of 39 respondents] (Figure 1B). Compared with the U.S. adult population, participants were more likely to report wheeze in the last 12 months (SMR 2.0; 95% CI: 1.6–2.4), and having physician-diagnosed current asthma (SMR 1.4; 95% CI: 1.0–1.9) (Table S1). Six (16%) of 38 participants with current asthma reported their asthma was diagnosed after they started working at the coffee roasting and packaging facility (Table 2). Participants were not more likely to report exertional dyspnea (SMR 1.0; 95% CI: 0.7–1.2) or cough (SMR 0.9; 95% CI: 0.6–1.4) compared with the U.S. adult population. Participants were more likely to report phlegm for three consecutive months or more in the last 12 months (SMR 1.9; 95% CI 1.4–2.5). Systemic symptoms (flu-like achiness, fever or chills, or unusual tiredness) in the last 12 months were reported by 52% of participants; 13% of participants reported their systemic symptoms were work-related.

Nearly all (96%) spirometry testing met criteria for acceptability and repeatability. Most (95%) participants had normal spirometry (Table S2). Of those with abnormal spirometry, seven had an obstructive pattern, nine had a restrictive pattern, and two had a mixed pattern; 16 of 18 participants with abnormal spirometry underwent bronchodilator testing. Seven of nine with obstructive or mixed pattern had bronchodilator testing and five of these seven (71%) participants had a significant improvement in FEV_1_ and one (14%) had a significant improvement in FVC. Two participants with severe airways obstruction improved with bronchodilator; one reported pre-existing lung disease prior to employment. Two of three participants with mild obstructive pattern and one of two with moderate obstruction improved with bronchodilator. One participant with a moderate mixed pattern that did not improve following bronchodilator administration was referred to a pulmonologist and diagnosed with obliterative bronchiolitis following an extensive diagnostic evaluation. This participant worked at a coffee production facility for 7 years during which time his job included adding flavoring to coffee; this case is detailed separately (44). The other participant with moderate mixed pattern improved with bronchodilator. Of the nine participants with mild restriction, seven had bronchodilator testing and none had a significant improvement in FEV1 or FVC.

Participants' mean percent predicted FEV_1_ was 102.3% (range: 39.8–141.1%), mean percent predicted FVC was 103.7% (range: 71.2–143.2), and mean FEV_1_/FVC ratio was 80.9% (range: 29.3–99.7%) (Table S2). SMRs for abnormal spirometry patterns were not elevated; restrictive patterns were less prevalent compared with the U.S. adult population (SMR 0.4; 95% CI 0.2–0.8) (Table S1). Thirty-three (9%) participants had elevated FeNO; participants who reported current asthma (*n* = 38) had an average FeNO of 44 ppb compared with 25 ppb for participants who did not report current asthma (*n* = 339) (*P* < 0.05). Participants who reported current asthma had lower percent predicted FEV_1_ (96.2 vs. 103.0%, *P* = 0.004) and FEV_1_/FVC (75.8 vs. 81.4, *P* < 0.0001) than participants without current asthma; there was no difference in percent predicted FVC (103.8 vs. 103.7%; *P* = 0.98).

We compared symptoms, diagnoses, and lung function parameters between participants who reported working near flavoring (*n* = 44) and participants who did not work near flavoring (*n* = 340) (Table S3). Participants who worked near flavoring reported more exertional dyspnea (24 vs. 14%; *P* = 0.08) and asthma attacks (14 vs. 6%; *P* = 0.08) than participants who did not work near flavoring. Usual cough was reported by 14% of participants who worked near flavoring and 10% of participants who did not work near flavorings (*P* = 0.47). Participants who worked near flavoring reported more chronic bronchitis (7 vs. 1%; *P* = 0.02) and ever receiving a diagnosis of asthma (30 vs. 15%; *P* = 0.03) than participants who did not work near flavoring. There were no substantial differences between the two groups in lung function parameters.

We compared symptoms, diagnoses, lung function parameters, and job tasks between participants categorized as atopic (*n* = 119) and participants categorized as non-atopic (*n* = 265) (Table S4). Atopic participants more often reported upper respiratory symptoms, problems smelling, phlegm, exertional dyspnea, trouble breathing, lower respiratory symptoms, and systemic symptoms than non-atopic participants. Atopic participants more often reported gastroesophageal reflux disease and ever receiving a diagnosis of asthma than non-atopic participants. There were no substantial differences in lung function parameters. Atopic participants had a higher mean FeNO than non-atopic participants (31 vs. 25 ppb; *P* = 0.04). More atopic participants reported working with green coffee beans than non-atopic participants (48 vs. 33%; *P* = 0.02).

Symptoms and lung function were similar between participants who worked at flavoring and non-flavoring facilities in our study (Table 3). Compared with previous investigations of flavoring-exposed workers, participants in our study had a lower prevalence of exertional dyspnea, cough, and obstruction, and a higher average percent predicted FEV_1_. The prevalence of wheeze was comparable with those observed in other flavoring-exposed populations. All study populations included only current workers and no former workers.

**Table 3:** Comparison of characteristics for participants from non-flavoring coffee facilities and flavoring coffee facilities to published findings from workers in the sentinel coffee facility, sentinel microwave popcorn facility, and three other microwave popcorn facilities.

	**12 non-flavoring coffee facilities (*n* = 227)**	**5 flavoring coffee facilities (*n* = 157)**	Sentinel flavoring coffee facility^*^(1, 18) (*n* = 75)	**Sentinel microwave popcorn facility^†^^‡^(7, 42) (*n* = 122)**	**3 other microwave popcorn facilities(7, 40, 41, 43) (*n* = 397)**
Exertional dyspnea (%)	14	17	28	26	26
Wheeze (%)	28	20	20	36	23
Cough (%)	9	13	16	24	24
% predicted FEV_1_	102.3	102.5	97.6	90.0	94.2
Obstruction (%)	2 (4 of 214)	2 (3 of 158)	7 (5 of 69)	10 (12 of 16)	4 (17 of 395)
Reversible (%)	100 (3 of 3)	50 (1 of 2)	33 (1 of 3)	11 (1 of 9)	31 (5 of 16)
**Mean TWA personal exposure parts per billion (range)**					
Diacetyl	7.3 (0.1–40.5)	24.9 (0.1–420.9)	57.7 (4.3–166.0)	19,938 (479–147,170)	328.4 (ND−2,740)
2, 3-pentanedione	4.5 (0.1–27.1)	19.1 (0.2–275.9)	46.1 (<5.2–199.0)	Not measured	Not measured

* *Symptoms reported by work area including grinding/packaging, flavoring, and roasting*.

† *Symptoms reported by job categories including ever mixer, never mixer, packaging non-isolated tanks, and packaging isolated tanks*.

‡ *Area sampling from mixing and packaging areas. ND, Not Detected*.

The mean TWAs measured for diacetyl and 2,3-pentanedione at the 12 non-flavoring facilities in our study were 7.3 and 4.5 ppb, respectively; the mean TWAs at the five flavoring facilities were 24.9 and 19.1 ppb, respectively (Table 3). The mean TWAs measured at the sentinel coffee facility that also flavored were 57.7 ppb for diacetyl and 46.1 ppb for 2,3-pentanedione. The mean TWA for diacetyl at the sentinel microwave popcorn production facility was 19,938 ppb.

## Discussion

Our findings demonstrate a burden of respiratory and mucous membrane abnormalities among workers at 17 coffee roasting and packaging facilities. Respiratory abnormalities were characterized by upper respiratory symptoms and wheeze, with a work-related pattern for some participants. The symptoms reported by participants might not represent a single work-related lung disease or condition. Rather, we observed a spectrum of symptoms that could indicate different occupational respiratory diseases including work-related asthma and obliterative bronchiolitis. Our findings likely represent overlapping effects of the different respiratory hazards in coffee roasting and packaging facilities we evaluated, which included diacetyl and 2,3-pentanedione sources, and other potential hazards such as green coffee bean and other dust.

We found evidence of severe lung disease in only a few workers. Spirometric abnormalities were only present in 5% of those studied, and two classic symptoms of obliterative bronchiolitis, exertional dyspnea and cough, were not in excess. Most symptoms and spirometric parameters were similar for participants who worked near flavoring and those who did not work near flavoring. However, one participant who added flavoring to coffee was diagnosed with obliterative bronchiolitis following referral to a pulmonologist; no cases of obliterative bronchiolitis were identified among workers who did not use flavorings. In addition, we measured higher alpha-diketone exposures in facilities that added flavorings compared with those that did not add flavorings. Thus, risk might be higher in facilities using flavorings, but data are limited. Other limitations are that we studied only current workers, and do not have information about the health status of former workers and whether any left employment because of lung disease. Furthermore, tenure among participants was relatively low, with a median of <3 years. Inclusion of former workers and longitudinal evaluation of longer-tenure workers in facilities that do not add flavorings could help account for the healthy worker effect and shed light on the longer-term risk of naturally occurring diacetyl in these settings.

Our findings are consistent with a burden of work-related asthma among participants. Past studies have demonstrated that coffee roasting and packaging workers were at an increased risk for work-related asthma (23, 28). We found more participants reported asthma than expected compared with the U.S. adult population. Lower respiratory symptoms, many of which are common symptoms of asthma, were frequently reported among participants and frequently with a work-related pattern, suggesting work-related asthma. Green coffee dust was frequently reported as a cause or exacerbator of lower respiratory symptoms. Although our study was not designed to investigate the underlying mechanism causing asthma, green coffee bean dust could have acted as a sensitizer in some workers (1). FeNO was elevated in nearly one in 10 workers, which can be an indication of eosinophilic airways inflammation or poorly controlled asthma (39). Six of the nine participants who had obstructive or mixed pattern on spirometry had significant improvement in FEV_1_ post-bronchodilator administration; this would be expected in uncontrolled asthma, and is a higher percentage than a recent study where roughly one-third of adults aged 40 years or older with obstruction improved post-bronchodilator (45).

Upper respiratory symptoms were the most commonly reported symptoms, often with a work-related pattern; eye symptoms were also commonly reported. Nose and eye symptoms were reported more than expected compared with the U.S. adult population. Participants overwhelmingly described dust as the cause or aggravation of their upper respiratory symptoms. Upper respiratory disease such as allergic rhinitis and sinusitis are sometimes associated with lower respiratory symptoms and asthma and might precede the diagnosis of asthma (46–50). Thus, controlling exposures associated with upper respiratory symptoms could ultimately serve to reduce the risk of asthma among coffee workers.

Atopic participants reported more upper and lower respiratory symptoms than non-atopic participants; atopic participants also reported more asthma. Upper respiratory inflammation (e.g., rhinitis, sinusitis) can result in suboptimal control of asthma (48, 49). Interestingly, atopic participants reported working more with green coffee beans. Green coffee dust is thought to be a more potent allergen than roasted coffee dust because roasting destroys some of the allergenic activity (51). N95 disposable filtering-face pieces might prevent symptoms related to green coffee dust and chaff, although are not protective against diacetyl and 2,3-pentanedione, which would require organic vapor cartridges (52).

Participants reported nearly twice as much wheeze than expected compared with the U.S. adult population, some with a work-related pattern; wheeze was the only lower respiratory symptom reported more than expected for those we could compare to the U.S. adult population. Wheeze is a common symptom of both obliterative bronchiolitis and asthma, and we cannot determine the underlying cause of wheeze among participants in our evaluation. The overlapping effects of different respiratory hazards in roasting and packaging facilities including asthmagens and alpha-diketones likely contributed to the increased risk of wheeze observed among participants.

Compared with workers studied from the sentinel coffee roasting and packaging facility where five cases of obliterative bronchiolitis were identified among former employees, and workers from microwave popcorn production facilities, our population had lower prevalences of exertional dyspnea, cough, and spirometric abnormality likely reflecting a lower risk of obliterative bronchiolitis. The sentinel microwave popcorn facility had fewer participants with reversible spirometric obstruction (11%) than would be expected, perhaps reflecting the greater relative importance of obliterative bronchiolitis as an adverse respiratory health outcome in that setting (45).

Mean diacetyl and 2,3-pentanedione levels from the facilities that flavored in our study were more than three times higher than those from facilities that did not flavor, but still less than half of those measured from the sentinel coffee roasting and packaging facility. However, measurements at the sentinel coffee facility were likely underestimates of exposure to former workers due to improved ventilation and different flavoring formulation that were implemented before sampling occurred (18). Mean diacetyl levels measured from flavoring facilities in our study were far below those from the popcorn facilities where the risk of flavoring-related obliterative bronchiolitis was first described. These exposure differences indicate that for the coffee facilities we studied, particularly those that did not flavor, the risk of obliterative bronchiolitis is lower than the historical risk associated with the microwave popcorn industry and likely also the sentinel coffee facility. Nonetheless, flavoring and non-flavoring facilities in our study had TWAs above the NIOSH recommended exposure limit (REL) for both diacetyl (5 ppb) and 2,3-pentanedione (9.3 ppb) (52).

Our study has several limitations. First, the medical surveys only included current workers; we did not capture former workers who could have work-related health effects. If former workers were included, the prevalence of respiratory symptoms or disease might have been higher; some workers might have left employment prior to our surveys due to work-related respiratory symptoms or disease. Thus, our results were likely influenced by the healthy worker effect, a potential bias caused by workers choosing work environments with lower exposure or leaving work (53). Also, the facilities we evaluated did not have known health concerns before our evaluations and were mostly at the request of management and thus might not have been representative of other settings in the industry. In addition, the participation rate was lower than desired and our findings may not be representative of all workers at these facilities. Finally, our medical surveys were not intended to be diagnostic evaluations; we did not evaluate for airway hyperreactivity or for variability in pulmonary function at and away from work, nor did we assess for Immunoglobulin E (IgE) sensitization to green coffee bean or other workplace allergens. In addition, we did not assess for findings such as air trapping in high-resolution computed tomography (HRCT), which might be more sensitive for small airways disease.

Our findings are not intended to be representative of the entire coffee roasting and packaging industry because of the variation in production processes, including the amount of coffee produced, use of flavoring, size of workforce, automaticity, and use of engineering controls; we observed this large variation in the production processes in the 17 facilities we evaluated. Despite the limitations, this study is one of largest evaluations of coffee roasting and packaging facilities. Combining evaluations from 17 facilities allowed us to evaluate the burden of respiratory abnormalities in a group of coffee roasting and packaging workers.

The burden of respiratory abnormalities we observed, including a range of upper and lower respiratory symptoms, likely reflects the effects of workplace exposures. Our findings indicate occupational respiratory health concerns among coffee roasting and packaging workers are not limited to obliterative bronchiolitis or specific to facilities that use flavorings. The symptoms and patterns we found likely represent the overlapping health effects of different respiratory hazards facing coffee roasting and packaging workers, including green coffee bean and other dust, diacetyl, 2,3-pentanedione, and potentially other respiratory hazards. Public health authorities should be aware of the different potential respiratory health hazards in coffee roasting and packaging facilities, including flavoring and non-flavoring facilities. Healthcare providers should be aware that workers at coffee roasting and packaging facilities are potentially at risk for several occupational respiratory diseases with potentially overlapping symptoms and functional manifestations, including work-related asthma and obliterative bronchiolitis.

## Data Availability Statement

Due to restrictions imposed under the US privacy act and the limitations of what participants consented to, the data underlying the analyses presented, beyond what is provided in the paper, are confidential and not available to researchers outside the National Institute for Occupational Safety and Health (NIOSH). For more information about NIOSH's policy regarding sensitive data, see https://www.cdc.gov/niosh/ocas/datahandle.html.

## Ethics Statement

The NIOSH Institutional Review Board reviewed and approved this study (NIOSH Protocol 17-RHD-06XP). All participants provided their written informed consent to participate.

## Author Contributions

RH, EF-L, RB, KF, MV, RN, JC-G, and KC contributed conception and design of the study. NE and KF organized the database. RH, EF-L, KF, and NE performed the statistical analyses. RH wrote the first draft of the manuscript. All authors contributed to manuscript revision, read, and approved the submitted version.

### Conflict of Interest

The authors declare that the research was conducted in the absence of any commercial or financial relationships that could be construed as a potential conflict of interest.
