# Supplementary material for: The Burden of Respiratory Abnormalities Among Workers at Coffee Roasting and Packaging Facilities
Source: Front Public Health. 2020 Jan 30;8:5. doi: 10.3389/fpubh.2020.00005 (PMC7003510; doi:10.3389/fpubh.2020.00005)
Supplement: Supplementary file 1 [file Data_Sheet_1.docx]

**Table S1:** Standardized morbidity ratios for reported symptoms, self-reported diagnoses, and spirometric abnormalities, for workers in 17 coffee roasting and production facilities, N=376*

| Health condition | Observed Number | Expected Number | SMR  (95% CI)† |
| --- | --- | --- | --- |
| Symptoms |  |  |  |
| Watery, itchy eyes last 12 months | **173** | **144.1** | **1.2 (1.0-1.4)** |
| Stuffy, itchy, or runny nose last 12 months | **239** | **197.8** | **1.2 (1.1-1.4)** |
| Sinus problems last 12 months | 103 | 121.5 | 0.8 (0.7-1.0) |
| Phlegm 3 consecutive month or more | **40** | **21.5** | **1.9 (1.4-2.5)** |
| Wheeze last 12 months | **92** | **46.6** | **2.0 (1.6-2.4)** |
| Exertional dyspnea | 59 | 61.4 | 1.0 (0.7-1.2) |
| Cough 3 consecutive months or more | 18 | 20.3 | 0.9 (0.6-1.4) |
| Diagnoses |  |  |  |
| Chronic bronchitis (physician-diagnosed) | **5** | **15.9** | **0.3 (0.1-0.7)** |
| Ever asthma (physician-diagnosed) | 65 | 53.2 | 1.2 (<1.0-1.6) |
| Current asthma (physician-diagnosed) | **38** | **27.6** | **1.4 (1.0-1.9)** |
| Spirometry (N = 359)* |  |  |  |
| Obstruction (no mixed) | 7 | 12.1 | 0.6 (0.3-1.2) |
| Restriction (no mixed) | **9** | **21.0** | **0.4 (0.2-0.8)** |
| Mixed only | 2 | 2.7 | 0.7 (0.2-2.7) |

SMR = standardized morbidity ratio

*N based on participants included in comparisons due to incomplete survey or spirometry data

†Statistically significant 95% Confidence Intervals (CIs) shown in bold

**Table S2:** Spirometry acceptability and repeatability, results and mean
values for workers in 17 coffee roasting and production facilities, N=372*

| Spirometry acceptability and repeatability | n (%) |
| --- | --- |
| ≥3 acceptable trials, FVC† and FEV_1_‡ both within 150 mL | 356 (96%) |
| ≥2 acceptable trials, FVC and FEV_1_ both within 250 mL | 13 (3%) |
| Does not meet acceptability and/or repeatability criteria for FVC or FEV_1_ | 1 (<1%) |
| Not interpretable | 2 (1%) |
|  |  |
| Spirometry results | **n (%)** |
| Normal | 349 (95%) |
| Obstruction | 7 (2%) |
| Mild | 3 (1%) |
| Moderate | 2 (1%) |
| Severe | 2 (1%) |
| Restriction | 9 (2%) |
| Mild | 9 (2%) |
| Mixed | 2 (1%) |
| Moderate | 2 (1%) |
|  |  |
| Spirometry values | **Mean (range)** |
| % predicted FEV_1_ mean (range) | 102.3 (39.8–141.1) |
| % predicted FVC mean (range) | 103.7 (71.2–143.2) |
| FEV_1_/FVC mean (range) | 80.9 (29.3–99.7) |
| FEV_3_/FVC mean (range) | 94.3 (50.9–100) |
| %FEF25–75§ | 102.6 (11.4–209.9) |

*N based on participants included in comparisons due to incomplete survey or spirometry data

†Forced vital capacity

‡Forced expiratory volume in one second

§ Forced expiratory flow between 25% and 75%

**Table S3:** Comparison of reported symptoms, self-reported doctor diagnoses, and lung function parameters in workers near flavoring and workers not near flavoring for workers in 17 coffee roasting and production facilities, N=384

| Symptom(s) | Not near flavoring, n=340 | Near flavoring, n=44 | | P value |
| --- | --- | --- | --- | --- |
| Upper respiratory symptoms (reported at least one of the following) | 228 (67%) | 24 (55%) | | 0.11 |
| Nose symptoms* | 220 (65%) | 24 (55%) | | 0.19 |
| Sinusitis or sinus problems | 97 (29%) | 8 (18%) | | 0.13 |
| Eye symptoms† | 170 (50%) | 27 (61%) | | 0.15 |
| Problem with ability to smell | 40 (12%) | 6 (14%) | | 0.74 |
| Phlegm on most days for 3 months | 33 (10%) | 7 (16%) | | 0.23 |
| Lower respiratory symptoms | 158 (46%) | 21 (47%) | | 0.88 |
| Exertional dyspnea‡ | 48 (14%) | 11 (24%) | | 0.08 |
| Breathing trouble | 69 (20%) | 10 (23%) | | 0.71 |
| Chest wheezing or whistling | 81 (24%) | 13 (30%) | | 0.41 |
| Usual cough§ | 34 (10%) | 6 (14%) | | 0.47 |
| Awoke with chest tightness | 47 (14%) | 6 (14%) | | 0.97 |
| Asthma attack | 20 (6%) | 6 (14%) | | 0.08 |
| Awoke with shortness of breath | 23 (7%) | 5 (11%) | | 0.30 |
| Systemic symptoms (flu-like achiness; fever or chills; unusual tiredness or fatigue) | 174 (51%) | 25 (57%) | | 0.48 |
| Diagnosis |  |  |  | |
| Hay fever or nasal allergies | 78 (23%) | 10 (23%) | | 0.97 |
| Eczema | 40 (12%) | 7 (16%) | | 0.45 |
| Heart disease | 11 (3%) | 0 | | 0.10 |
| Gastroesophageal reflux disease | 24 (7%) | 6 (14%) | | 0.16 |
| Chronic bronchitis | **3 (1%)** | **3 (7%)** | | **0.02** |
| Emphysema | 1 (<1%) | 0 | | 0.62 |
| Chronic obstructive pulmonary disease | 1 (<1%) | 0 | | 0.62 |
| Vocal cord dysfunction | 1 (<1%) | 0 | | 0.62 |
| Asthma (ever) | **52 (15%)** | **13 (30%)** | | **0.03** |
| Asthma (still) | 31 (59%) | 7 (58%) | | 0.70 |
| Parameter |  |  | |  |
| % predicted forced expiratory volume in one second (FEV_1_) | 102.6 | 100.6 | | 0.39 |
| % predicted forced vital capacity (FVC) | 103.8 | 102.5 | | 0.53 |
| FEV_1_/FVC | 81.0 | 79.7 | | 0.30 |
| FEV_3_/FVC | 94.4 | 93.7 | | 0.40 |
| Exhaled nitric oxide (FENO) | 27.1 | 24.5 | | 0.51 |

Note: statistical significant differences shown in bold; “-“= A four week question or work-related question was not asked for the symptom.

*Nose symptoms includes one or both of the following: 1) stuffy, itchy, or runny nose or 2) stinging, burning nose.

†Eye symptoms includes one or both of the following: 1) watery, itchy eyes or 2) stinging, burning eyes.

‡ This question did not specifically ask about exertional dyspnea within the past 12 months; participants were asked, “Are you troubled by shortness of breath when hurrying on level ground or walking up a slight hill”

§This question did not specifically ask about a cough within the past 12 months; participants were asked, “Do you usually have a cough?” If the participants answered “yes”, they were then asked, “Have you had a cough at any time in the last 4 weeks?”

**Table S4:** Comparison of reported symptoms, self-reported doctor diagnoses, and lung function parameters in workers with and without atopy, for workers in 17 coffee roasting and production facilities, N=384

| Symptom(s) | Not atopic, n=265 | Atopic, n=119 | | P value |
| --- | --- | --- | --- | --- |
| Upper respiratory symptoms (reported at least one of the following) | 149 (56%) | **103 (87%)** | | **<0.01** |
| Nose symptoms* | **144 (54%)** | **100 (84%)** | | **<0.01** |
| Sinusitis or sinus problems | **44 (17%)** | **61 (51%)** | | **<0.01** |
| Eye symptoms† | **154 (58%)** | **43 (36%)** | | **<0.01** |
| Problem with ability to smell | **19 (7%)** | **27 (23%)** | | **<0.01** |
| Phlegm on most days for 3 months | **21 (8%)** | **19 (16%)** | | **0.02** |
| Lower respiratory symptoms | **107 (40%)** | **72 (61%)** | | **<0.01** |
| Exertional dyspnea‡ | **33 (12%)** | **26 (22%)** | | **0.02** |
| Breathing trouble | **38 (14%)** | **41 (34%)** | | **<0.01** |
| Chest wheezing or whistling | **44 (17%)** | **50 (42%)** | | **<0.01** |
| Usual cough§ | 23 (9%) | 17 (14%) | | 0.10 |
| Awoke with chest tightness | **30 (11%)** | **23 (19%)** | | **0.04** |
| Asthma attack | **7 (3%)** | **19 (16%)** | | **<0.01** |
| Awoke with shortness of breath | 16 (6%) | 12 (10%) | | 0.17 |
| Systemic symptoms (flu-like achiness; fever or chills; unusual tiredness or fatigue) | **117 (44%)** | **82 (69%)** | | **<0.01** |
| Diagnosis |  |  |  | |
| Heart disease | 8 (3%) | 3 (3%) | | 0.78 |
| Gastroesophageal reflux disease | **14 (5%)** | **16 (13%)** | | **0.01** |
| Chronic bronchitis | 2 (1%) | 4 (3%) | | 0.07 |
| Emphysema | 1 (<1%) | 0 | | 0.39 |
| Chronic obstructive pulmonary disease | 1 (<1%) | 0 | | 0.62 |
| Vocal cord dysfunction | 1 (<1%) | 0 | | 0.62 |
| Asthma (ever) | **27 (10%)** | **38 (32%)** | | **<0.01** |
| Asthma (still) | 12 (44%) | 26 (68%) | | >0.05 |
| Parameter |  |  | |  |
| % predicted forced expiratory volume in one second (FEV_1_) | 102.2 | 102.8 | | 0.69 |
| % predicted forced vital capacity (FVC) | 103.5 | 104.2 | | 0.60 |
| FEV_1_/FVC | 80.9 | 80.8 | | 0.94 |
| FEV_3_/FVC | 94.3 | 94.3 | | 0.93 |
| Exhaled nitric oxide (FENO) | **25.1** | **30.6** | | **0.04** |
| Current tasks |  |  | |  |
| Production | 234 (88%) | 99 (83%) | | 0.18 |
| Green beans | **74 (33%)** | **40 (48%)** | | **0.02** |
| Roast | 40 (18%) | 20 (24%) | | 0.25 |
| Grind | 114 (51%) | 48 (57%) | | 0.35 |
| Move beans | 136 (61%) | 49 (58%) | | 0.67 |
| Flavor (at facilities that flavor) | 15 (14%) | 8 (24%) | | 0.19 |
| Flavor (yes or no) | 15 (6%) | 8 (7%) | | 0.67 |
| Package | **169 (76%)** | **42 (50%)** | | **<0.01** |
| Clean | 136 (61%) | 41 (49%) | | 0.06 |
| Maintenance | 75 (34%) | 30 (36%) | | 0.73 |
| Quality control | 99 (37%) | 48 (38%) | | 0.58 |

Note: statistical significant differences shown in bold; “-“= A four week question or work-related question was not asked for the symptom.

*Nose symptoms includes one or both of the following: 1) stuffy, itchy, or runny nose or 2) stinging, burning nose.

†Eye symptoms includes one or both of the following: 1) watery, itchy eyes or 2) stinging, burning eyes.

‡ This question did not specifically ask about exertional dyspnea within the past 12 months; participants were asked, “Are you troubled by shortness of breath when hurrying on level ground or walking up a slight hill”

§This question did not specifically ask about a cough within the past 12 months; participants were asked, “Do you usually have a cough?” If the participants answered “yes”, they were then asked, “Have you had a cough at any time in the last 4 weeks?”
